# Bilateral Achilles Tendon Rupture in a Patient With End-Stage Chronic Obstructive Pulmonary Disease

**DOI:** 10.7759/cureus.93230

**Published:** 2025-09-25

**Authors:** Aye Moh Moh Paing, Aye Pyae Tin Hla, Thiri May Sin, Wai Yan Linn

**Affiliations:** 1 Medicine, Victoria Hospital, Kirkcaldy, GBR; 2 General Internal Medicine, Victoria Hospital, Kirkcaldy, GBR; 3 General Practice, Norfolk and Norwich University Hospital, Norwich, GBR

**Keywords:** achilles tendon rupture bilateral, atraumatic tendon rupture, copd and steroid use, corticosteroids, systemic corticosteroid

## Abstract

This case report describes an unusual case of bilateral Achilles tendon rupture in a 65-year-old patient with severe chronic obstructive pulmonary disease (COPD). The patient presented with decreased mobility, severe pain, and swelling in both calves following multiple courses of dexamethasone for the acute exacerbation of COPD. Notably, there was no history of trauma. Tendon rupture is a recognised, albeit rare, side effect of corticosteroid use. This case highlights the importance of counselling patients about the potential adverse effects of corticosteroids, including their impact on tendon health. It emphasises the need to use steroids judiciously in clinical practice and to avoid their use in patients with a history of atraumatic tendon rupture. Early involvement of the orthopaedic team is recommended to facilitate timely diagnosis, guide management decisions, and optimise functional recovery.

## Introduction

Achilles tendon rupture is a well-recognized musculoskeletal complication associated with long-term corticosteroid use, particularly in patients with chronic inflammatory conditions [1]. While unilateral ruptures are more common, bilateral atraumatic Achilles tendon rupture remains a rare and serious clinical presentation, often indicating underlying systemic factors or corticosteroid-related adverse effects [2].

Corticosteroids, widely used in the management of chronic obstructive pulmonary disease (COPD), are known to contribute to tendon degeneration through inhibition of collagen synthesis and reduced tendon vascularity [3]. These structural side effects - including osteoporosis and myopathy - can significantly impair musculoskeletal integrity, particularly in elderly patients with multiple comorbidities such as diabetes mellitus, chronic kidney disease, gout, rheumatoid arthritis, collagen disorders, and thyroid or parathyroid dysfunction. The risk is further heightened by medications such as systemic or locally injected corticosteroids, fluoroquinolones [4], and statins [5].

Management of Achilles tendon rupture includes both operative and nonoperative approaches. Surgical repair is typically reserved for younger or more active patients, while conservative treatment, such as functional bracing with a VACOped® boot (OPED Medical, Inc., Braselton, GA, USA), is often preferred in older or frail individuals.

This case report presents a 65-year-old man with end-stage COPD who developed bilateral Achilles tendon ruptures after receiving multiple courses of dexamethasone. Over a five-month period, the patient underwent five separate five-day courses of oral dexamethasone and was also maintained on inhaled corticosteroids. The absence of trauma and the temporal relationship with corticosteroid therapy highlight the importance of clinician awareness regarding the musculoskeletal risks of corticosteroids. The case also emphasises the need for a multidisciplinary approach in diagnosis and management, particularly in patients with limited physiological reserve.

## Case presentation

A 65-year-old gentleman with end-stage COPD presented with a one-week history of bilateral leg pain. He described a sudden sensation of a "pop" in both ankles, accompanied by significant difficulty walking and pain radiating from the back of both legs into the soles of his feet. He also reported nocturnal cramps, increasing calf swelling, and purple discoloration. This occurred two months after completing five separate courses of oral dexamethasone (8 mg daily for five days per course), administered over a five-month period for recurrent COPD exacerbations. There was no history of trauma or injury, raising suspicion of an adverse drug reaction or underlying systemic condition. According to local guidelines, he was treated with tetracyclines during most exacerbations and penicillin intermittently, with no history of quinolone use.

At presentation, his BMI was 22.3 (normal range: 18.5-24.9). There was significant tenderness in both calves and a palpable gap in the region of the Achilles tendons, suggesting possible rupture. Notably, haematoma was absent. The patient was unable to walk or stand on tiptoes. Additionally, the Thompson test was positive, with no plantarflexion of the ankles upon calf compression, strongly indicating Achilles tendon rupture.

**Figure 1:**
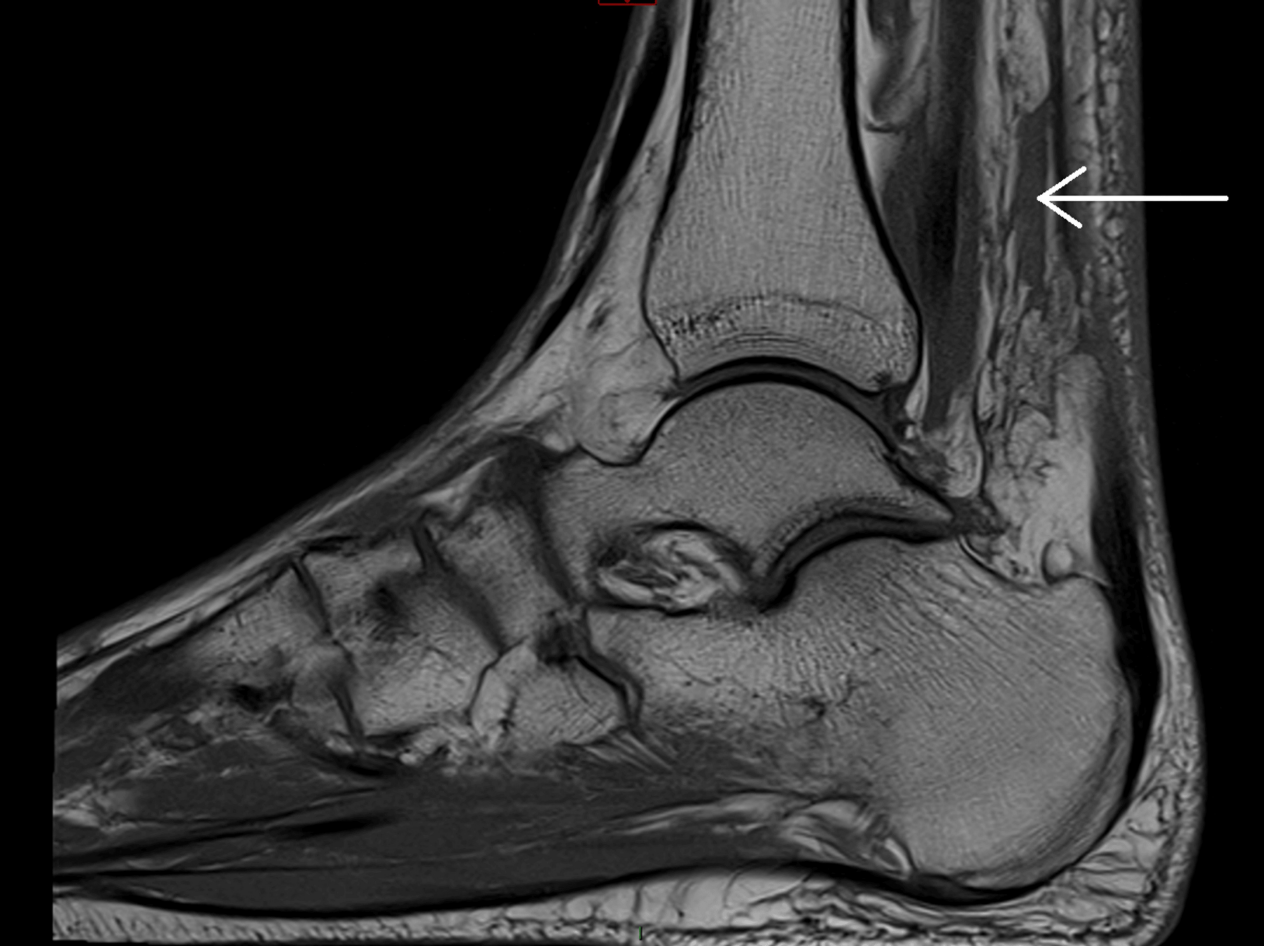
Left ankle MRI – 4 cm gap showing Achilles tendon rupture with local soft tissue inflammatory changes, indicated by white arrow

**Figure 2:**
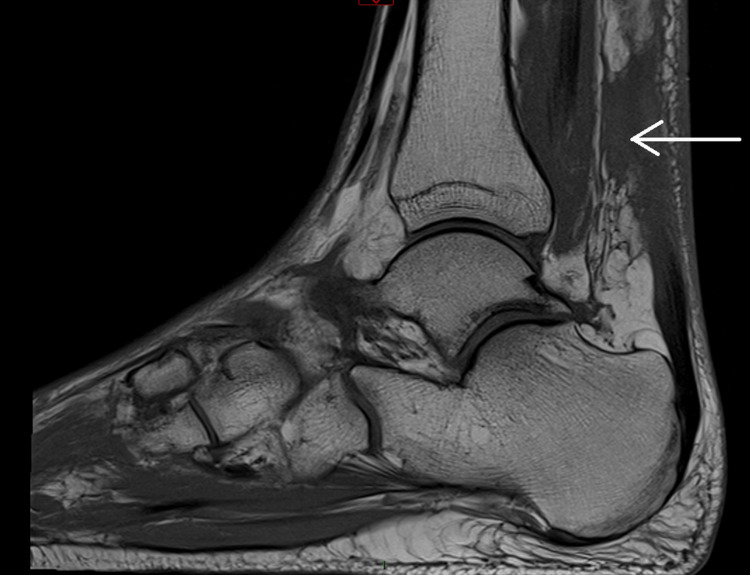
Left ankle MRI – 4 cm gap showing Achilles tendon rupture with local soft tissue inflammatory changes, indicated by white arrow

**Figure 3:**
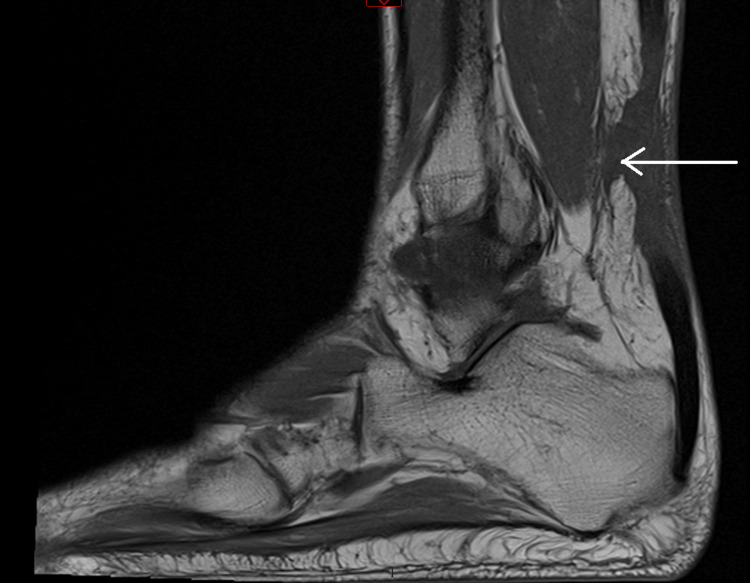
Right ankle MRI – 5 cm gap showing Achilles tendon rupture with local soft tissue inflammatory changes, indicated by white arrow

**Figure 4:**
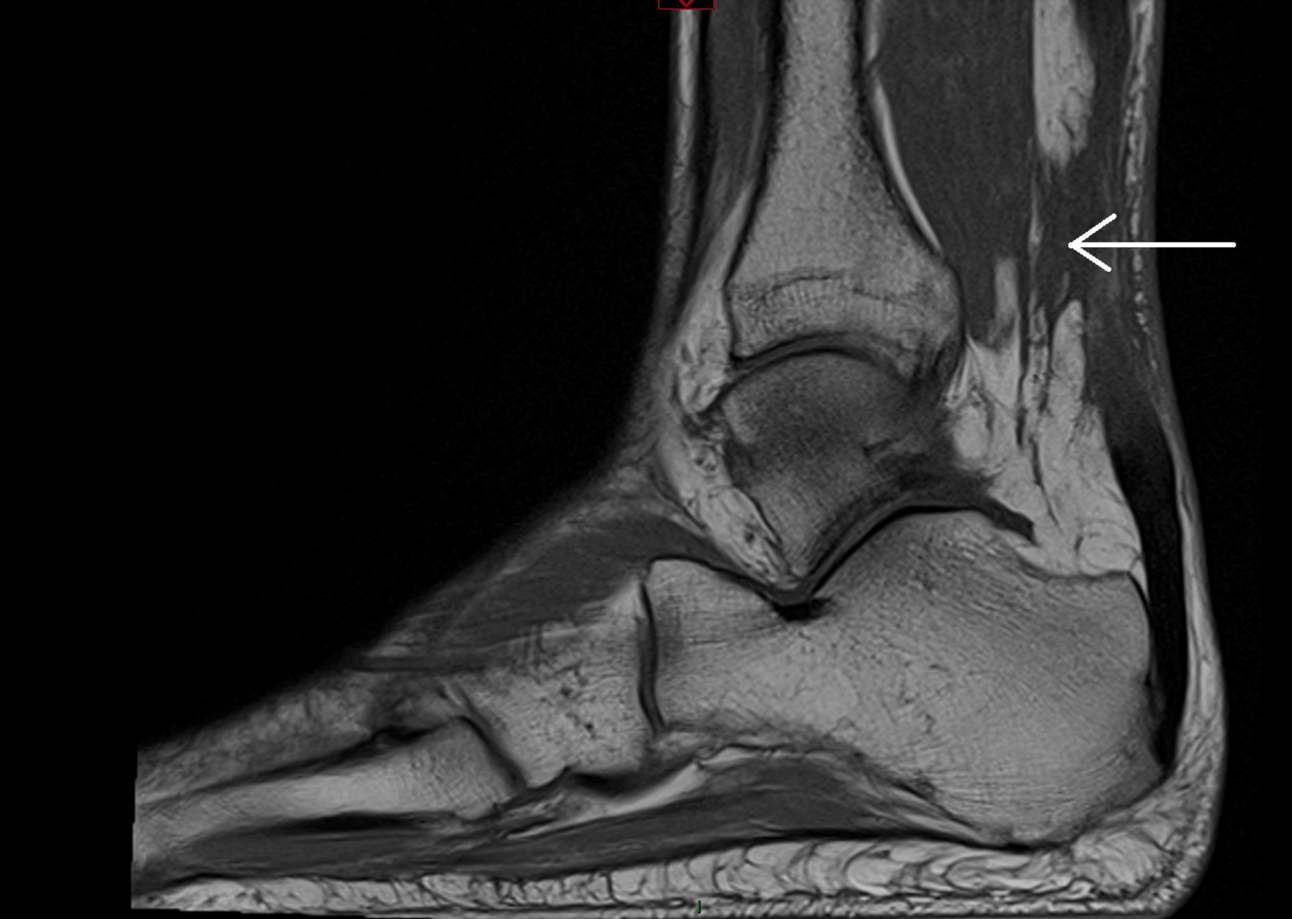
Right ankle MRI – 5 cm gap showing Achilles tendon rupture with local soft tissue inflammatory changes, indicated by white arrow

Following orthopaedic review and MRI imaging, bilateral Achilles tendon rupture was confirmed. The left ankle showed a 4 cm gap with associated soft tissue inflammatory changes, as demonstrated in sagittal views of non-contrast MRI (Figure 1 and Figure 2). The right ankle revealed a 5 cm gap with similar inflammatory changes (Figure 3 and Figure 4). Due to limited imaging resources at our district hospital, initial ultrasound was unsuccessful, necessitating non-contrast MRI.

Concurrent blood tests, including thyroid function, creatine kinase, and D-dimer, were performed to exclude alternative diagnoses such as endocrine disorders, myopathy, or deep vein thrombosis. All values were within normal limits, except for elevated cholesterol at 8 mmol/L (normal range: 3.3-6.5 mmol/L).

The tendon rupture was managed conservatively using VACOped walking boots, following a joint decision by the orthopaedic, respiratory, and rehabilitation teams, considering the patient’s comorbidities. He underwent a period of rehabilitation in a community hospital. However, his mobility remained poor due to limitations imposed by end-stage COPD, which hindered further progress. He remains wheelchair-dependent and can only manage short distances with a rollator due to exertional breathlessness.

## Discussion

Atraumatic bilateral Achilles tendon rupture is a rare condition. One of the major contributing factors is the use of corticosteroids. Other possible contributing factors include diabetes [6], xanthomatosis [7], chronic renal failure [8], and the use of fluoroquinolones [9,10].

It is imperative to obtain a comprehensive drug history for patients presenting with Achilles tendon rupture, particularly in the presence of comorbidities. This practice is essential for identifying potential risk factors-especially the need to avoid fluoroquinolones, which may exacerbate tendon pathology [10].

In this case, the patient had no significant past medical history apart from COPD, which led to multiple repeated courses of corticosteroid therapy. Corticosteroids are known to impair tendon integrity by inhibiting collagen synthesis and reducing vascularity, contributing to degeneration and increased rupture risk. Additionally, the patient was found to have a deranged lipid profile, specifically elevated cholesterol, which has been implicated in tendon pathology due to its effects on tendon homeostasis and healing [11].

In addition to corticosteroid exposure, hyperlipidemia may play a synergistic role in tendon degeneration. Elevated lipid levels have been associated with impaired collagen integrity, increased oxidative stress, and reduced tendon vascularity - all of which contribute to the pathogenesis of tendinopathy [11]. Corticosteroids, particularly when used repeatedly or systemically, are known to suppress collagen synthesis, impair tenocyte function, and reduce tendon vascularity, further weakening tendon structure [3,12]. When combined, these metabolic and pharmacologic factors may amplify the risk of tendon injury, especially in vulnerable populations such as the elderly or those with chronic inflammatory conditions [4].

Long-term dexamethasone use has been shown to decrease the expression of type I collagen in the Achilles tendon, weakening its structure and increasing susceptibility to rupture [12]. It also impairs the repair process of degenerated or partially ruptured tendons, increasing the likelihood of complete rupture even after minor stress [13].

One study reported that 10% of patients attending outpatient chest clinics for COPD were receiving long-term oral corticosteroids. Over a 12-year period, 10 cases of Achilles tendon rupture were documented, estimating a risk of one in 600 per year for these patients [14]. Bilateral Achilles tendon rupture without trauma is extremely uncommon. In this case, the patient received multiple short courses of oral dexamethasone rather than continuous long-term corticosteroid therapy. This highlights that even intermittent, high-dose systemic corticosteroid exposure - particularly when combined with maintenance inhaled corticosteroids - can significantly elevate the risk of tendon rupture [15]. The absence of fluoroquinolone use further supports corticosteroids as the most likely contributing factor in this atraumatic bilateral Achilles tendon rupture.

Therefore, judicious use of corticosteroids is essential, particularly when considering both systemic and inhaled forms. Clinicians should be aware of the potential for cumulative corticosteroid exposure from multiple sources, even when each course is brief or appears low risk individually. This case highlights the need for thorough patient counselling regarding the potential musculoskeletal risks of corticosteroid therapy, including short courses and inhaled formulations. Patients should be advised to promptly report new musculoskeletal symptoms, and clinicians should maintain a high index of suspicion for tendon injuries in at-risk individuals.

Management of Achilles tendon rupture includes both operative and nonoperative treatments. While operative treatment reduces the risk of re-rupture, it also increases the likelihood of complications such as infection. A systematic review and meta-analysis concluded that the final decision on the management of acute Achilles tendon ruptures should be based on patient-specific factors and shared decision-making [16].

In this case, the patient was managed conservatively with VACOped boots due to frailty and end-stage COPD. He was discharged after undergoing a period of physiotherapy at a rehabilitation hospital.

The functional bracing protocol at our hospital using the VACOped boot for Achilles tendon injuries involves a structured six-stage progression over approximately 16 weeks. Initially, the patient is placed in a below-knee back slab in full equinus (foot pointed downward) and remains non-weight bearing for the first two weeks. From weeks three to 10, the patient transitions through various locked positions of the VACOped boot - from 30° to 0° plantarflexion - gradually increasing mobility and weight bearing, starting with partial weight bearing using elbow crutches. Active plantarflexion exercises begin in week five. By weeks nine to 10, the boot is fully unlocked, and crutch use is reduced as tolerated. From week 11 onward, the boot is removed except in vulnerable situations, and the patient progresses from partial to full weight bearing under physiotherapy guidance, following Achilles tendon rehabilitation protocols. The boot or cast must be worn continuously during stages 1 to 6 unless otherwise advised.

This case emphasises the critical importance of prescribing corticosteroids judiciously and ensuring comprehensive patient education regarding potential complications. Clinicians must exercise vigilance, particularly in patients with chronic conditions such as COPD, which necessitate prolonged corticosteroid therapy.

## Conclusions

Bilateral Achilles tendon rupture in the absence of trauma is a rare but significant complication of systemic corticosteroid therapy. This case underscores the importance of recognising corticosteroid-induced tendon pathology, especially in patients with chronic illnesses such as COPD who are frequently exposed to high-dose or repeated steroid regimens

Clinicians should maintain a high index of suspicion for tendon injuries in patients presenting with acute heel pain and a history of corticosteroid use. Periodic musculoskeletal screening, at least through physical examination, is advisable for COPD patients receiving chronic or repeated corticosteroid therapy. Corticosteroids can weaken tendons and bones, increasing the risk of complications such as Achilles tendon rupture. Screening helps detect early signs of tendon or muscle pathology, particularly in patients with additional risk factors such as hyperlipidaemia or diabetes. A proactive approach may improve outcomes and prevent serious mobility impairments.

Early diagnosis and appropriate management-whether surgical or conservative-are essential to optimise functional outcomes and prevent further morbidity. A multidisciplinary approach involving physicians, orthopaedic surgeons, and rehabilitation specialists is crucial for comprehensive care.
